# Supplementary material for: Quantitative Protein Topography Measurements by High Resolution Hydroxyl Radical Protein Footprinting Enable Accurate Molecular Model Selection
Source: Sci Rep. 2017 Jul 3;7:4552. doi: 10.1038/s41598-017-04689-3 (PMC5495787; doi:10.1038/s41598-017-04689-3)
Supplement: Supplementary file 1 — Supplementary Information [file 41598_2017_4689_MOESM1_ESM.pdf]

Quantitative Protein Topography Measurements by High Resolution Hydroxyl Radical Protein Footprinting Enable Accurate Molecular Model Selection

Supplementary Information

Boer Xie[1], Amika Sood[1], Robert J. Woods[1] and Joshua S. Sharp[2]

[1]Complex Carbohydrate Research Center, Department of Chemistry, University of Georgia, Athens, GA 30602;

[2]Department of BioMolecular Sciences, School of Pharmacy, University of Mississippi, Oxford, MS 38655

Table 1. A list of oxidized amino acids from lysozyme and myoglobin with slope, k/k_p, SASA and $SASA_{GXG}$

Residues	Slope (±SD)	k/k_p	SASA (±SD) (Å^2)	$SASA_{GXG}$ (Å^2)
Lys C6	11.8 (1.46)	29.2	39.25 (6.1)	102.3
Lys C30	2.05 (0.49)	29.2	1.26 (0.1)	102.3
Lys C64	0.79 (0.17)	29.2	0 (3.26)	102.3
Lys M105	2.69 (0.4)	20.5	0.09 (1.89)	158.3
Lys W62	2.05 (0.25)	17.4	116.64 (8.31)	224.6
Lys W63	0.51 (0.08)	17.4	33.92 (10.63)	224.6
Lys W108	0.8 (0.12)	17.4	10.35 (3.3)	224.6
Lys W111	0.56 (0.06)	17.4	24.99 (2.23)	224.6
Lys W123	0.95 (0.05)	17.4	71.04 (5.65)	224.6
Lys Y20	0.48 (0.05)	12	57.76 (7.36)	193.1
Lys Y23	0.4 (0.12)	12	50.35 (2.84)	193.1
Lys Y53	0.11 (0.01)	12	16.03 (3.84)	193.1
Lys F3	0.009 (0.002)	11.2	10.96 (8.74)	180.1
Lys F34	0.25 (0.05)	11.2	49.17 (2.84)	180.1
Lys F38	0.074 (0.005)	11.2	17.75 (8.74)	180.1
Lys H15	0.16 (0.02)	9.3	23.7 (13.39)	154.6
Lys I124	0.21 (0.02)	4.4	22.74 (5.84)	147.3
Lys R68	0.17 (0.05)	2.9	103.69 (2.12)	195.5
Lys K1	0.033 (0.007)	2.2	62.61 (9.51)	164.5
Lys K33	0.085 (0.004)	2.2	57.43 (0.13)	164.5
Lys V2	0.025 (0.002)	1.9	103.49 (6.63)	122.3
Lys V109	0.3 (0.018)	1.9	116.38 (1.7)	122.3
Lys V120	0.04 (0.004)	1.9	22.11 (8.1)	122.3
Lys T47	0.007 (0.0007)	1.6	158.66 (5.28)	106.2
Lys T51	0.004 (0.0008)	1.6	1.29 (0.67)	106.2
Lys S50	0.011 (0.003)	1.4	0.73 (2.23)	77.4
Lys Q41	0.019 (0.002)	0.66	104.42 (0.47)	141.2
Lys Q121	0.069 (0.008)	0.66	76.45 (5.93)	141.2
Lys N37	0.06 (0.008)	0.44	93.03 (1.42)	114.3
Lys N39	0.017 (0.004)	0.44	42.04 (3.71)	114.3
Lys D52	0.004 (0.0009)	0.42	30.31 (1.4)	113
Lys A107	0.118 (0.02)	0.14	27.08 (7.07)	64.9
Lys A110	0.092 (0.01)	0.14	7.58 (6.71)	64.9
Lys A122	0.044 (0.005)	0.14	31.19 (7.67)	64.9
Myo M131	1.06 (0.12)	20.5	0.25 (0.44)	158.3
Myo W7	0.29 (0.06)	17.4	23.94 (4.13)	224.6
Myo W14	0.22 (0.03)	17.4	6.13 (3.44)	224.6
Myo F33	0.04 (0.004)	11.2	1.37 (1.24)	180.1

Myo F123	0.04 (0.006)	11.2	1.08 (0.95)	180.1
Myo F138	0.05 (0.01)	11.2	12.32 (3.29)	180.1
Myo F151	0.26 (0.03)	11.2	34.61 (5.13)	180.1
Myo H24	0.015 (0.001)	9.3	3.64 (2.78)	154.6
Myo H36	0.33 (0.08)	9.3	53.46 (9.56)	154.6
Myo H113	1.14 (0.08)	9.3	72.29 (8.16)	154.6
Myo H116	1.04 (0.04)	9.3	68.79 (13.02)	154.6
Myo H119	0.43 (0.12)	9.3	51.98 (7.86)	154.6
Myo I21	0.12 (0.007)	4.4	39.59 (5.99)	147.3
Myo I30	0.22 (0.02)	4.4	23.23 (3.41)	147.3
Myo L11	0.073 (0.02)	4.4	36.7 (7.27)	146.2
Myo L40	0.070 (0.002)	4.4	15.7 (3.02)	146.2
Myo L69	0.044 (0.01)	4.4	0.13 (0.28)	146.2
Myo L72	0.054 (0.01)	4.4	7.58 (2.42)	146.2
Myo L137	0.12 (0.002)	4.4	50.24 (7.28)	146.2
Myo L149	0.22 (0.007)	4.4	46.7 (8.54)	146.2
Myo R31	0.011 (0.001)	2.9	101.98 (10.11)	195.5
Myo R139	0.003 (0.0002)	2.9	30.79 (6.51)	195.5
Myo K42	0.033 (0.007)	2.2	71.35 (6.74)	164.5
Myo K77	0.028 (0.008)	2.2	104.43 (25.52)	164.5
Myo K147	1.07 (0.08)	2.2	133.25 (13.63)	164.5
Myo V13	0.093 (0.02)	1.9	8.29 (4.27)	122.3
Myo V67	0.037 (0.008)	1.9	112.93 (8.21)	122.3
Myo V68	0.18 (0.03)	1.9	49.05 (5.19)	122.3
Myo T66	0.008 (0.0009)	1.6	38.6 (6.8)	106.2
Myo T70	0.16 (0.06)	1.6	91.2 (6.33)	106.2
Myo P37	0.054 (0.02)	1	79.27 (6.59)	105.2
Myo P120	0.43 (0.12)	1	72.52 (3.88)	105.2
Myo E18	0.073 (0.006)	0.69	66.05(13.18)	141.2
Myo E27	0.019 (0.001)	0.69	33.58 (9.72)	141.2
Myo E38	0.031 (0.002)	0.69	118.37 (16.26)	141.2
Myo E136	0.016 (0.003)	0.69	87.29 (9.02)	141.2
Myo Q8	0.2 (0.04)	0.66	131.89 (16.07)	143.7
Myo Q9	0.2 (0.06)	0.66	66.87 (7.59)	143.7
Myo Q26	0.019 (0.004)	0.66	10.34 (5.01)	143.7
Myo Q130	0.22 (0.03)	0.66	3.11 (1.25)	143.7
Myo N12	0.1 (0.03)	0.44	76.35 (4.27)	114.3
Myo D126	0.098 (0.01)	0.42	91.93 (6.78)	113
Myo A125	0.21 (0.05)	0.14	74.93 (2.55)	64.9

Table 2. A list of amino acids found to be oxidized from both denatured and native lysozyme and myoglobin with slope, k/k_P, SASA and $SASA_{GXG}$

Residues	Slope $_D$ (±SD)	Slope $_N$ (±SD)	k/k_P	SASA (±SD) ($Å^2$)	SASA $_{GXG}$ ($Å^2$)
LysW62	3.32 (0.9)	2.05 (0.25)	17.4	116.64 (8.31)	224.6
LysW63	2.58 (0.7)	0.51 (0.08)	17.4	33.92 (10.63)	224.6
LysW108	5.31 (0.31)	0.8 (0.12)	17.4	10.35 (3.3)	224.6
LysW111	1.6 (0.14)	0.56 (0.06)	17.4	24.99 (2.23)	224.6
LysW123	2.98 (0.17)	0.95 (0.05)	17.4	71.04 (5.65)	224.6
LysY20	0.85 (0.06)	0.48 (0.05)	12	57.76 (7.36)	193.1
LysY23	0.93 (0.07)	0.4 (0.12)	12	50.35 (2.84)	193.1
LysY53	0.19 (0.03)	0.11 (0.01)	12	16.03 (3.84)	193.1
LysF3	0.15 (0.007)	0.009 (0.002)	11.2	10.96 (8.74)	180.1
LysF34	0.44 (0.02)	0.25 (0.05)	11.2	49.17 (5.53)	180.1
LysF38	0.23 (0.02)	0.074 (0.005)	11.2	17.75 (8.74)	180.1
LysH15	0.54 (0.23)	0.16 (0.02)	9.3	23.7 (13.39)	154.6
LysI124	0.66 (0.04)	0.21 (0.02)	4.4	22.74 (5.84)	147.3
LysV109	0.22 (0.03)	0.3 (0.018)	1.9	116.38 (1.7)	122.3
LysN37	0.085 (0.004)	0.06 (0.008)	0.44	76.93 (7.07)	114.3
LysN39	0.071 (0.002)	0.017 (0.004)	0.44	42.04 (3.71)	114.3
MyoW7	1.9 (0.28)	0.29 (0.06)	17.4	23.94 (4.13)	224.6
MyoW14	1.56 (0.4)	0.22 (0.03)	17.4	6.13 (3.44)	224.6
MyoF33	0.80 (0.001)	0.04 (0.004)	11.2	1.37 (1.24)	180.1
MyoF138	0.15 (0.04)	0.05 (0.01)	11.2	12.32 (3.29)	180.1
MyoF151	1.34 (0.33)	0.26 (0.03)	11.2	34.61 (5.13)	180.1
MyoH24	0.46 (0.11)	0.015 (0.001)	9.3	3.64 (2.78)	154.6
MyoH36	0.54 (0.1)	0.33 (0.08)	9.3	53.46 (9.56)	154.6
MyoH119	0.53 (0.09)	0.43 (0.12)	9.3	51.98 (7.86)	154.6
MyoI21	0.15 (0.04)	0.12 (0.007)	4.4	39.59 (5.99)	147.3
MyoL11	0.13 (0.01)	0.073 (0.02)	4.4	36.7 (7.27)	146.2
MyoL40	0.16 (0.02)	0.070 (0.002)	4.4	15.7 (3.02)	146.2
MyoL72	0.43 (0.09)	0.054 (0.01)	4.4	7.58 (2.42)	146.2
MyoL137	0.16 (0.04)	0.12 (0.002)	4.4	50.24 (7.28)	146.2
MyoL149	1.14 (0.19)	0.22 (0.007)	4.4	46.7 (8.54)	146.2
MyoR31	0.028 (0.004)	0.011 (0.001)	2.9	101.98 (10.11)	195.5
MyoR139	0.024 (0.004)	0.003 (0.0002)	2.9	30.79 (6.51)	195.5
MyoK42	0.039 (0.01)	0.033 (0.007)	2.2	71.35 (6.74)	164.5
MyoV13	0.13 (0.027)	0.093 (0.02)	1.9	8.29 (4.27)	122.3
MyoV68	0.34 (0.07)	0.18 (0.03)	1.9	49.05 (5.19)	122.3
MyoE27	0.039 (0.01)	0.019 (0.001)	0.69	33.58 (9.72)	141.2
MyoE38	0.031 (0.005)	0.031 (0.002)	0.69	118.37 (16.26)	141.2
MyoE136	0.018 (0.002)	0.016 (0.003)	0.69	87.29 (9.02)	141.2

MyoQ26	0.008 (0.001)	0.019 (0.004)	0.66	10.34 (5.01)	143.7
MyoN12	0.12 (0.005)	0.1 (0.03)	0.44	76.35 (4.27)	114.3

Table 3. A list of details of the homology models generated using SWISS MODEL

PDB ID	Sequence Coverage	Sequence Identity	All-Atom RMSD (Å)
1LZE	1.00	99.2	1.2
2GV0	1.00	69.0	1.6
2BQJ	0.98	59.8	1.8
2Z2E	0.99	50.8	2.4
4L41	0.95	36.9	3.1
3CB7	0.93	37.5	3.8
1GD6	0.92	42.9	4.2
1IIZ	0.92	38.7	4.3
2RSC	0.92	42.9	4.6

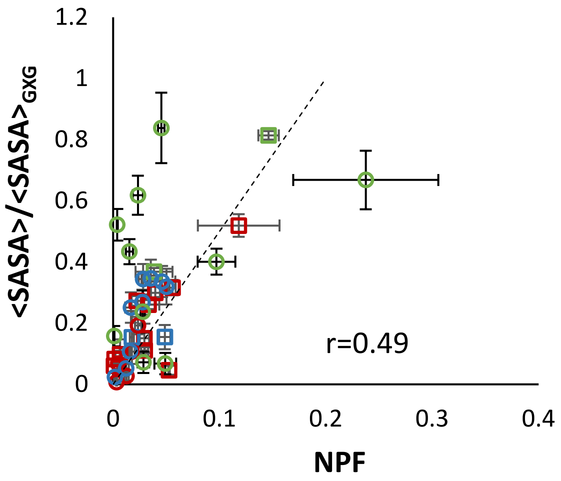

Supplementary Figure 1. Correlating the fractional <SASA> from the native fold and normalizing by free amino acid reactivity based on the same set of amino acids as the correlation study using native : denatured data from myoglobin (circles) and lysozyme (squares). Red – highly reactive residues; Blue – moderately reactive residues; and Green – poorly reactive residues. Data shown from triplicate experiments using mean ± SD plotted for all data (x-axis error bars), with fractional <SASA> from MD simulation of the protein structure ± SD from the MD trajectory (y-axis error bars).

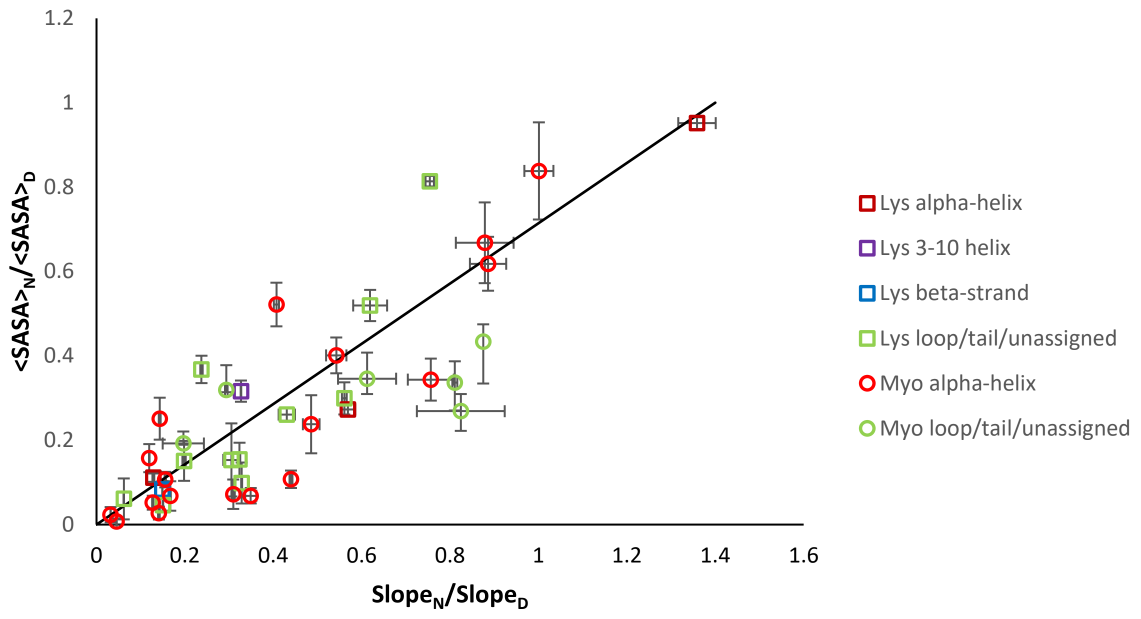

Supplementary Figure 2. Correlation between multi-point HR-HRPF data by comparing reactivities in denatured form versus native form and fractional <SASA> based on all non-sulfur containing residues from myoglobin (circles) and lysozyme (squares). Each data point represents an oxidized amino acid identified in both denatured and native sample. Red – α-helix; Purple – 3-10 helix; Blue: β-strand; Green – unstructured or unassigned secondary structure.

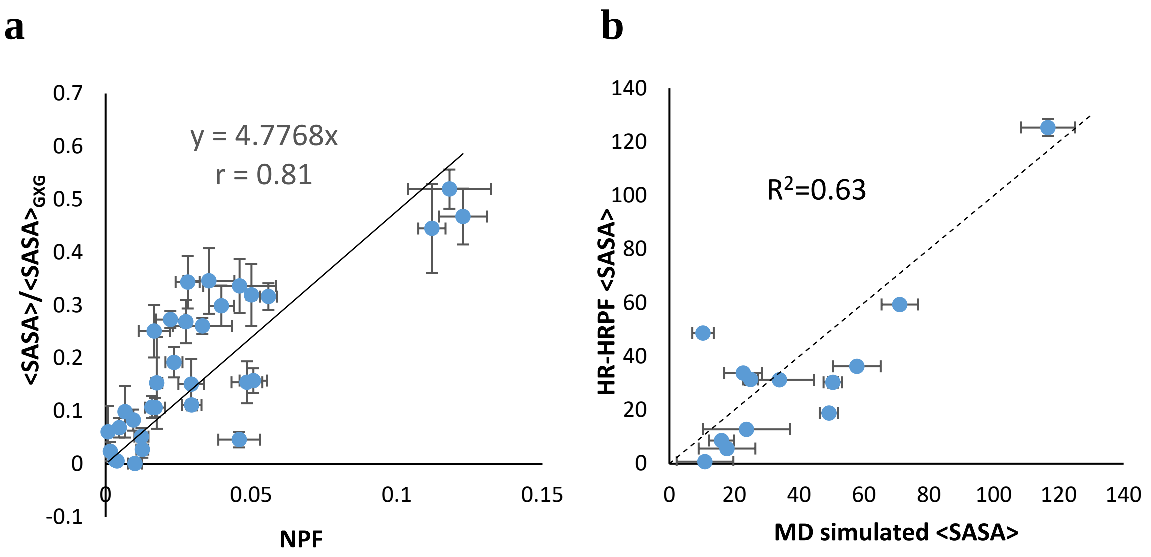

Supplementary Figure 3. Evaluation of <SASA> prediction regression models based on NPFs calculated from multi-point HR-HRPF data. (**a**) Dual protein-based linear regression model made based on multi-point HR-HRPF data from all non-sulfur containing residues with k/ kp > 4 from both myoglobin and lysozyme. (**b**) Comparison between predicted lysozyme <SASA> by HR-HRPF and actual lysozyme <SASA> from MD simulation using the dual protein predictive model. Mean ± SD plotted for all data from triplicate experiments, and R^2 is calculated against the model y=x.

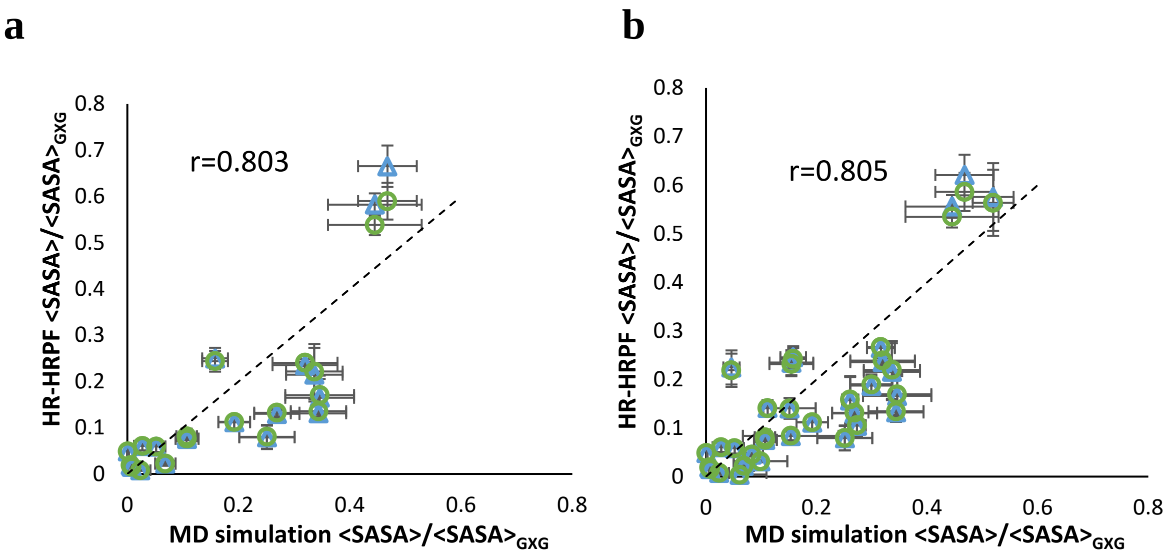

Supplementary Figure 4. Robustness test for regression models based on NPFs obtained from all non-sulfur containing residues with k/ kp >4 by comparing overall model and jackknife leave-one-out model. (a). Single protein-based model (myoglobin) and (b). Dual protein-based model (both myoglobin and lysozyme). Data shown from triplicated experiments. Mean ± SD plotted for all data. Pearson correlation coefficients (r) shown in the figure was calculated based on fractional <SASA> calculated from MD simulation and fractional <SASA> derived from HR-HRPF data of jackknife test. △ – Jackknife model; ○ – Overall model.

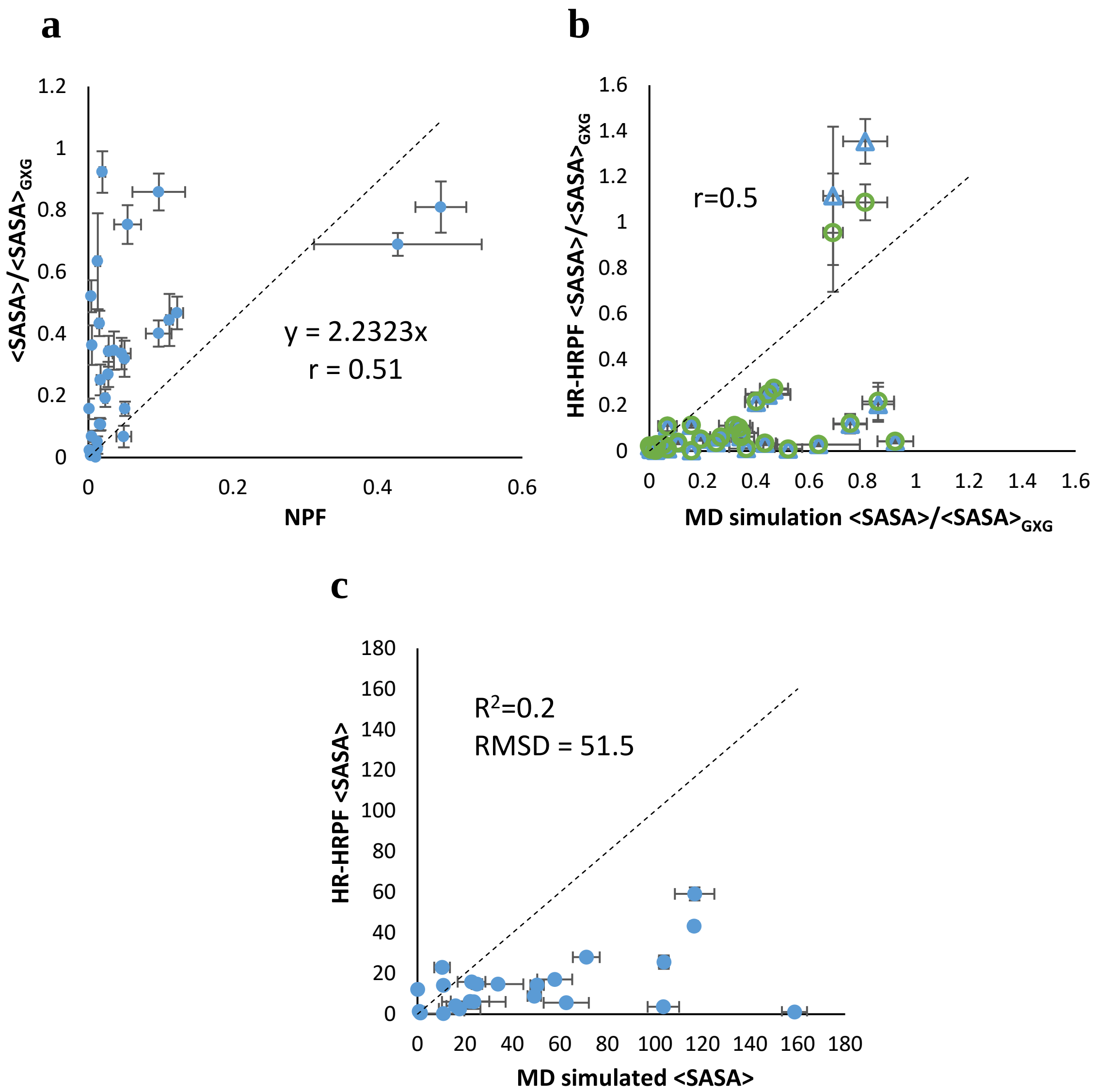

Supplementary Figure 5. Evaluation of single protein-based linear regression model made using all non-sulfur containing residues with $k/k_p>1$. (a). Linear regression model using multi-point HR-HRPF data from myoglobin; (b). Comparison of overall model and jackknife model of the proposed linear regression model for robustness test (△ – Jackknife model; ○ – Overall model); and (c). Comparison of lysozyme <SASA> calculated from HRPF data and actual lysozyme <SASA> from MD simulation. Mean ± SD plotted for all data from triplicate experiments.

a **b**

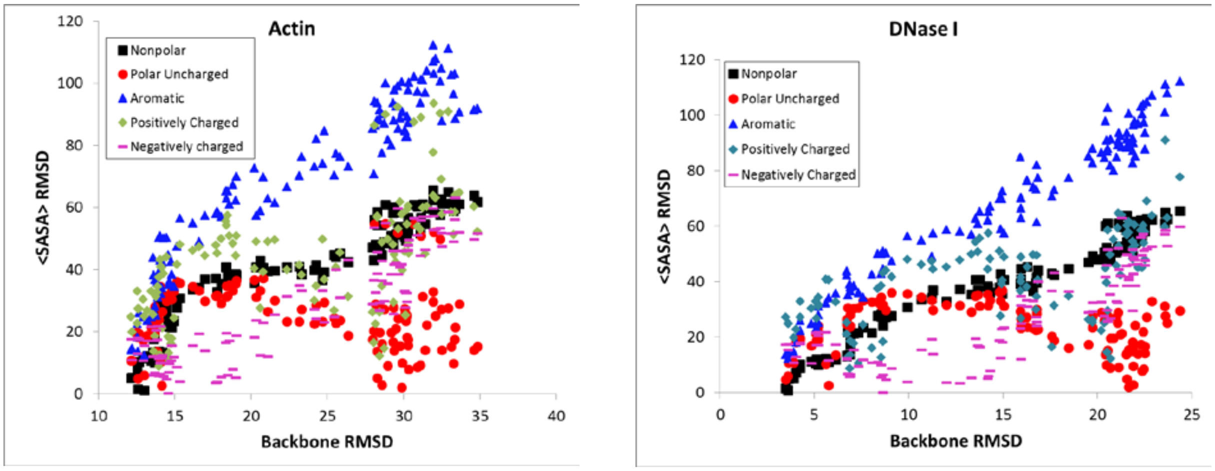

Supplementary Figure 6. Effectiveness of <SASA> of different categories of amino acids as constraints for molecular simulations. (**a**) actin, and (**b**) DNase I.

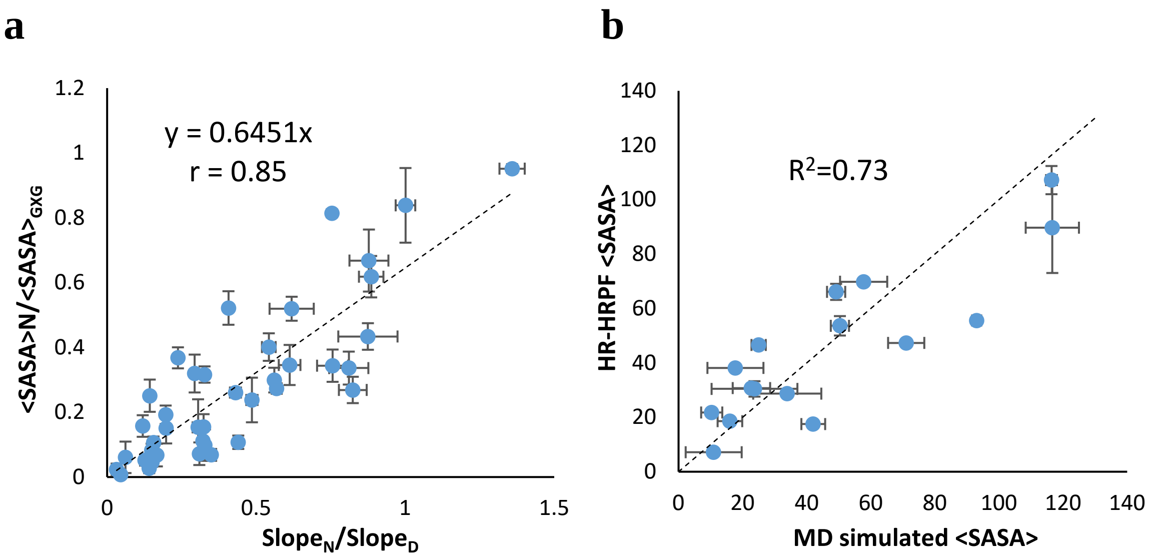

Supplementary Figure 7. Evaluation of <SASA> prediction regression models based on HR-HRPF data by comparing reactivities in denatured form versus native form. (**a**) Dual protein-based linear regression model made based on multi-point HR-HRPF data from all non-sulfur containing residues from both myoglobin and lysozyme. (**b**) Comparison between predicted lysozyme <SASA> by HRPF and actual lysozyme <SASA> from MD simulation. Mean ± SD plotted for all data from triplicated experiments, and R^2 is calculated against the model y=x.

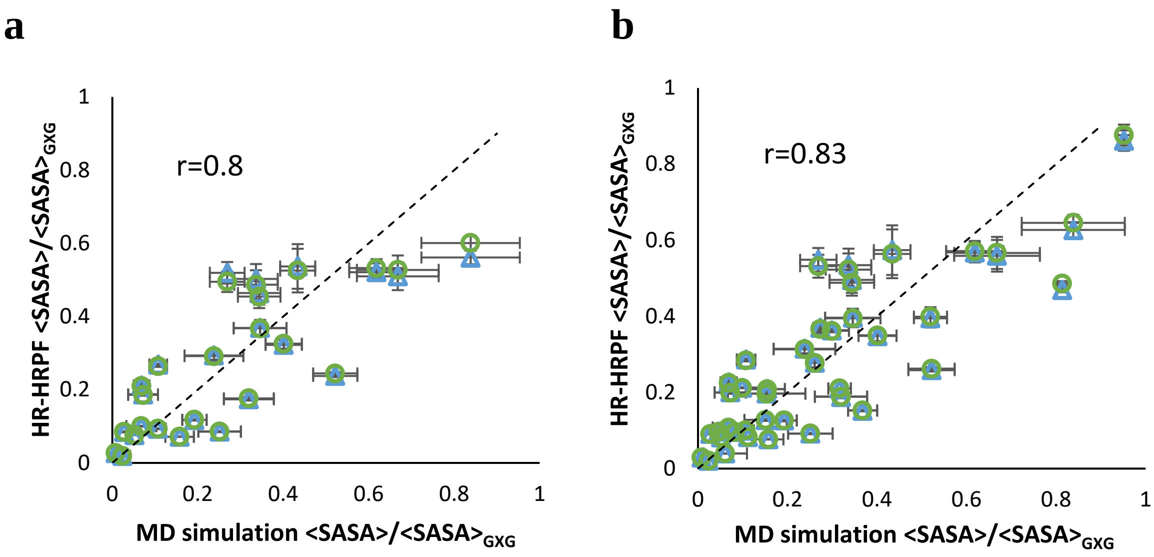

Supplementary Figure 8. Robustness test for regression models based on the ratio of reactivity (native : denatured) of all non-sulfur containing residues by comparing overall model and jackknife model. (a). Single protein-based model (myoglobin) and (b). Dual protein-based model (both myoglobin and lysozyme). Data shown from triplicated experiments. Mean ± SD plotted for all data. Pearson correlation coefficients (r) shown in the figure was calculated based on fractional <SASA> calculated from MD simulation and fractional <SASA> derived from multi-point HR-HRPF data of jackknife test. △ – Jackknife model; ○– Overall model.

Note 1: Peptide Level and Residue Level Quantification

Peptide level quantitation of the average oxidation events per peptide is calculated by summing the ion intensities of all the oxidized peptide masses multiplied by the number of oxidation events required for the mass shift (e.g., one event for +16, two events for +32, etc.), then divided by the sum of the ion intensities of all unoxidized and oxidized peptide masses (eq S1):

$$\mathrm{P} = \frac{I(+16)_{oxidized}*1+I(+32)_{oxidized}*2+I(+48)_{oxidized}*3+\cdots}{I_{unoxidized}+I(+16)_{oxidized}+I(+32)_{oxidized}+I(+48)_{oxidized}+\cdots} \quad \text{(S1)}$$

where P denotes the average oxidation events per peptide and I denotes the intensities of unoxidized and oxidized peptides.

Identification and quantification of each oxidation site at residue level was carried out using the ETD spectra collected from LC-MS/MS runs. The actual fractional oxidation of a given sequence ion is defined as the ratio between the oxidized sequence ion intensity to the sum of the intensity of the corresponding oxidized and unoxidized sequence ion (eq S2):

$$f(FI_i)_{actual} = \frac{\sum I(FI_i)_{oxidized}}{\sum I(FI_i)_{oxidized} + \sum I(FI_i)_{unoxidized}} \quad \text{(S2)}$$

where f $(FI_i)_{actual}$ denotes the fractional oxidation of c or z ion *i*; $I(FI_i)$ denotes the intensity of the c or z ion *i*, whether the unoxidized and oxidized form. The absolute level of oxidation for a given amino acid *i* is based on both the average oxidation event of peptide and the fractional oxidation of the corresponding sequence ions (eq S3):

$$oxidation/residue_i = P[\int(FI_i)_{actual} - \int(FI_{i-1})_{actual}] \quad \text{(S3)}$$

where P is the average oxidation events per peptide as derived from eq S1, and the term in square brackets is the actual fractional oxidation difference of two adjacent residues.
